# Ventricular synchrony is not significantly determined by absolute myocardial perfusion in patients with chronic heart failure: A ^13^N-ammonia PET study

**DOI:** 10.1007/s12350-018-01507-9

**Published:** 2018-11-15

**Authors:** Luis Eduardo Juarez-Orozco, Andrea G. Monroy-Gonzalez, Friso M. van der Zant, Nick Hoogvorst, Riemer H. J. A. Slart, Remco J. J. Knol

**Affiliations:** 1 Department of Nuclear Medicine and Molecular Imaging, University Medical Center Groningen, Hanzeplein 1, P.O. Box 30001, 9700RB Groningen, The Netherlands; 2 Turku PET Centre, Turku University Hospital and University of Turku, Kiinamyllynkatu 4-8, 20520 Turku, Finland; 3 Cardiac Imaging Division Alkmaar, Department of Nuclear Medicine, Northwest Clinics, Alkmaar, The Netherlands; 4 Department of Biomedical Photonic Imaging, TechMed Centre, University of Twente, Enschede, The Netherlands

**Keywords:** Positron emission tomography, myocardial perfusion, mechanical synchrony, heart failure

## Abstract

**Background:**

It is thought that heart failure (HF) patients may benefit from the evaluation of mechanical (dys)synchrony, and an independent inverse relationship between myocardial perfusion and ventricular synchrony has been suggested. We explore the relationship between quantitative myocardial perfusion and synchrony parameters when accounting for the presence and extent of fixed perfusion defects in patients with chronic HF.

**Methods:**

We studied 98 patients with chronic HF who underwent rest and stress Nitrogen-13 ammonia PET. Multivariate analyses of covariance were performed to determine relevant predictors of synchrony (measured as bandwidth, standard deviation, and entropy).

**Results:**

In our population, there were 43 (44%) women and 55 men with a mean age of 71 ± 9.6 years. The SRS was the strongest independent predictor of mechanical synchrony variables (*p* < .01), among other considered predictors including: age, sex, body mass index, smoking, diabetes mellitus, dyslipidemia, hypertension, rest myocardial blood flow (MBF), and myocardial perfusion reserve (MPR). Results were similar when considering stress MBF instead of MPR.

**Conclusions:**

The existence and extent of fixed perfusion defects, but not the quantitative PET myocardial perfusion parameters (sMBF and MPR), constitute a significant independent predictor of ventricular mechanical synchrony in patients with chronic HF.

**Electronic supplementary material:**

The online version of this article (10.1007/s12350-018-01507-9) contains supplementary material, which is available to authorized users.

## Introduction

Heart failure (HF) represents a major issue with an estimate of 26 million patients worldwide.^1^ Treatment advances in acute coronary syndromes and an aging population have steadily shifted the burden in cardiovascular disease profile through a substantial increase in the number of patients at risk of developing progressive HF.^2^ One of the main causes of HF is coronary artery disease (CAD), and CAD-related ischemic damage links to progressive maladaptive changes and abnormal ventricular function due to an altered myocardial architecture and adverse remodeling.^3^

HF research may benefit from techniques that provide evaluation of ventricular mechanical synchrony as well as from the evaluation of its determinants. On the one hand, because dyssynchrony may represent an earlier marker of a deteriorating ventricular function that conveys prognostic value for risk stratification,^4^ and on the other, because current (suboptimal) selection of HF patients for cardiac resynchronization therapy (CRT) mainly depends on ECG-QRS complex analysis (which evaluates electrical [dys]synchrony).^5^,^6^ As a matter of fact, relevant reports have documented an important and sustained proportion of CRT non-responders^7^,^8^ and it is considered that elements such as the imperfect link between measured electrical and mechanical synchrony, and the etiology of HF may factor into this phenomenon.

Quantitative PET myocardial perfusion imaging has robustly demonstrated its value in the evaluation of suspected ischemia in patients with and without history of cardiovascular events,^9^,^10^ and notably, is able to simultaneously provide complementary information on ventricular function during peak hyperemia based on the analysis of ECG-gated datasets.^11^ Such assessment of ventricular function extends beyond left ventricular ejection fraction into measurements of mechanical synchrony through phase analysis.^12^ Recently, ventricular mechanical synchrony has been implied as a useful marker for the detection of multivessel CAD,^13^ and an independent inverse relationship between quantitative myocardial perfusion and PET ventricular synchrony has been suggested.^14^

Notably, the link between quantitative PET myocardial perfusion and ventricular mechanical synchrony when considering the potential confounding influence of fixed perfusion defects, indicators of previous myocardial infarction [MI] and scarring, has not been investigated in HF patients. Hence, the present study aimed to explore the relationship between quantitative PET myocardial perfusion and peak stress ventricular mechanical synchrony when accounting for the presence and extent of pre-existing fixed perfusion defects in patients with chronic HF.

## Methods

### Patient Population

We retrospectively analyzed data from 98 patients with chronic HF referred for Nitrogen-13 ammonia PET/CT imaging for suspected myocardial ischemia and followed in the Northwest Clinics in Alkmaar, the Netherlands. Demographic and clinical characteristics including sex, age, body mass index (BMI), as well as cardiovascular risk factors including arterial hypertension (HTN), dyslipidemia, smoking status, type 2 diabetes mellitus (DM), history of previous myocardial infarction (MI), and baseline HF descriptors (suspected etiology, LVEF category and baseline NT-proBNP) were extracted from the electronic file system.

All patients gave written informed consent for use of their anonymous data for scientific purposes. Besides the standard imaging protocol and clinical management, no additional measurements or actions affecting the patient were performed. The study was approved by the institutional research department and approval of the local ethical committee for the present study was not necessary since the study does not fall within the scope of the Dutch Medical Research Involving Human Subjects Act (section 1.b WMO, February 26, 1998).

### PET Imaging

Every patient underwent a two-phase (rest and adenosine stress) PET scan using Nitrogen-13 ammonia as the perfusion radiotracer. All image data were acquired in list mode on a Siemens Biograph-16 TruePoint PET/CT (Siemens Healthcare, Knoxville, USA) with the TrueV option (axial field of view, 21.6 cm). This 3D system consists of a 16-slice CT and a PET scanner with four rings of lutetium oxyorthosilicate (LSO) detectors. Patients were instructed to fast overnight and to avoid the consumption of methylxanthine-, caffeine-containing beverages, or medications for 24 hours before the study. Image acquisition parameters and scanning protocol were previously described in detail.^15^

### Quantitative Perfusion

Based on the dynamic subsets, left ventricular contours were assigned automatically using the SyngoMBF software (Siemens Medical Solutions, Erlangen, Germany) with minimal observer intervention when appropriate. With a previously described 2-compartment kinetic model for Nitrogen-13 ammonia, stress and rest flow values in mL/g/minute were computed for each sample on the polar map through the resulting time-activity curves for global quantification.^16^ Myocardial perfusion reserve (MPR) was calculated as the ratio between the MBF during stress (sMBF) and MBF during rest (rMBF) and therefore expressed adimensionally. The total MPR, sMBF, and rMBF were calculated within the left ventricular region regionally according to the main vascular territories (LAD, LCx, and RCA).

### Semi-Quantitative Perfusion and Fixed Perfusion Defects

Pre-existing fixed perfusion defects were evaluated through semi-quantitative image analysis using the standard American Heart Association 17-segment model^17^ and a 5-point scale scoring system^18^ in order to calculate the summed rest score (SRS). The SRS was employed as a surrogate for the presence and extent of perfusion defects related to myocardial scarring.

### Left Ventricular Mechanical Synchrony

ECG-gated hyperemic stress images were analyzed with the QGS software package (Research Edition, PET Processing plugin, Cedars-Sinai, Los Angeles, CA, USA).^19^ Short-axis images were processed for ventricular edges and cavity volumes for each re-binned frame reconstructed for the average cardiac cycle. From the phase analysis, which allows for the evaluation of coincidence and uniformity of onset of wall motion,^20^ three previously described measurements of mechanical synchrony during stress were considered, namely bandwidth (BW), standard deviation (SD), and entropy (E).^21^

Briefly, BW conveys the time range that includes 95% of elements in the phase distribution, while SD corresponds to the standard deviation of the phase distribution. Thereon, E reflects the uniformity of the onset and progression of wall motion, expressed as a percentage. These measurements are understood as inversely proportional to ventricular synchrony and uniformity of contraction.

### Statistical Analysis

All continuous variables were described as means ± SD, while categorical variables were expressed as frequencies and percentages. Univariate analyses were conducted through independent ANOVAs in order to evaluate trend differences in ventricular mechanical synchrony variables (BW, SD, and E) across binned tertiles of quantitative PET perfusion variables (MPR and sMBF). Follow-up pairwise comparisons were corrected using the Bonferroni post hoc test.

Prior evaluation of the biserial correlations between BW, SD, and E (utilizing Pearson’s correlation coefficient), two sequential (stepwise) multivariate analyses of covariance (MANCOVA) were performed including sex, age, BMI, HTN, dyslipidemia, DM, smoking status, rMBF, and MPR *in the first model*, with the addition of SRS *in the second model*, as independent (i.e., predictor) variables, while BW, SD, and E were simultaneously input as the dependent (i.e., outcome) variables. Pearson’s correlation coefficient was used to examine for and discard collinearity of predictors. Independent significance of the evaluated predictors was assessed through Pillai’s trace criterion with an approximate *F* statistic.^14^ Effect sizes for the evaluated predictors (Eta squared [η2]) were reported alongside *p* values for which < .05 was considered statistically significant. Multivariate analyses were repeated utilizing sMBF (instead of MPR) as the quantitative perfusion variable. Complementarily, all analyses were repeated in a per-vessel territory analysis.

All statistical analyses were performed in SPSS (Released 2013. IBM SPSS Statistics for Windows, Version 22.0. Armonk, NY: IBM Corp., USA).

## Results

Baseline characteristics of the study sample of patients with chronic HF are shown in Table 1. There were 43 women and 55 men with a mean age of 72 ± 9.5 and 71 ± 9.6 years old, respectively. There was a substantial proportion of patients with HTN (45%) and roughly a quarter of the studied sample known with diabetes mellitus. Conversely, there was a low prevalence of active smokers (6%). There were 59% and 41% of patients with reduced and preserved LVEF, respectively.

**Table 1:** Baseline population characteristics

Variables	Summary
Demographics—mean (st dev)	
Age	71 (9.6)
Women (%)	43 (44)
BMI (Kg/m^2^)	28 (4.3)
Risk factors and CV/HF history—*n* (%)	
HTN	44 (45)
Dyslipidemia	29 (26)
DM	24 (25)
Smokers	6 (6)
History of previous MI	39 (40)
Ischemic HF	50 (51)
Hypertensive cardiomyopathy	17 (17)
Idiopathic HF	19 (19)
NYHA classification	
I-II	60 (6)
II	54 (55)
III	30 (31)
IV	8 (8)
Reduced LVEF	42 (43)
LVEF—mean (st dev)	33 (8)
Mid-range LVEF	16 (16)
LVEF—mean (st dev)	45 (9)
Preserved LVEF	40 (41)
LVEF—mean (st dev)	63 (8)
NT-proBNP (pg/mL)—mean (st dev)	483.4 (718.6)
Quantitative perfusion parameters—mean (st dev)	
Rest MBF (mL/g/min)	0.93 (0.29)
Stress MBF (mL/g/min)	1.72 (0.63)
MPR	1.92 (0.61)
Semi-quantitative perfusion—mean (st dev)	
SRS	10 (8.5)
Ventricular mechanical synchrony—mean (st dev)	
BW (ms)	56 (39.5)
SD (ms)	15.8 (11.8)
E (%)	45 (12.9)

*BMI*, body mass index; *BW*, bandwidth; *CV*, cardiovascular, *DM*, type 2 diabetes mellitus; *E*, entropy; *HF*, heart failure; *HTN*, arterial hypertension; *MBF*, myocardial blood flow; *MI*, myocardial infarction; *MPR*, myocardial perfusion reserve; *SD*, standard deviation; *SRS*, summed rest score

Mean quantitative PET MPR was below the generally applied pathological threshold of 2.0.^22^

**Figure 1:**
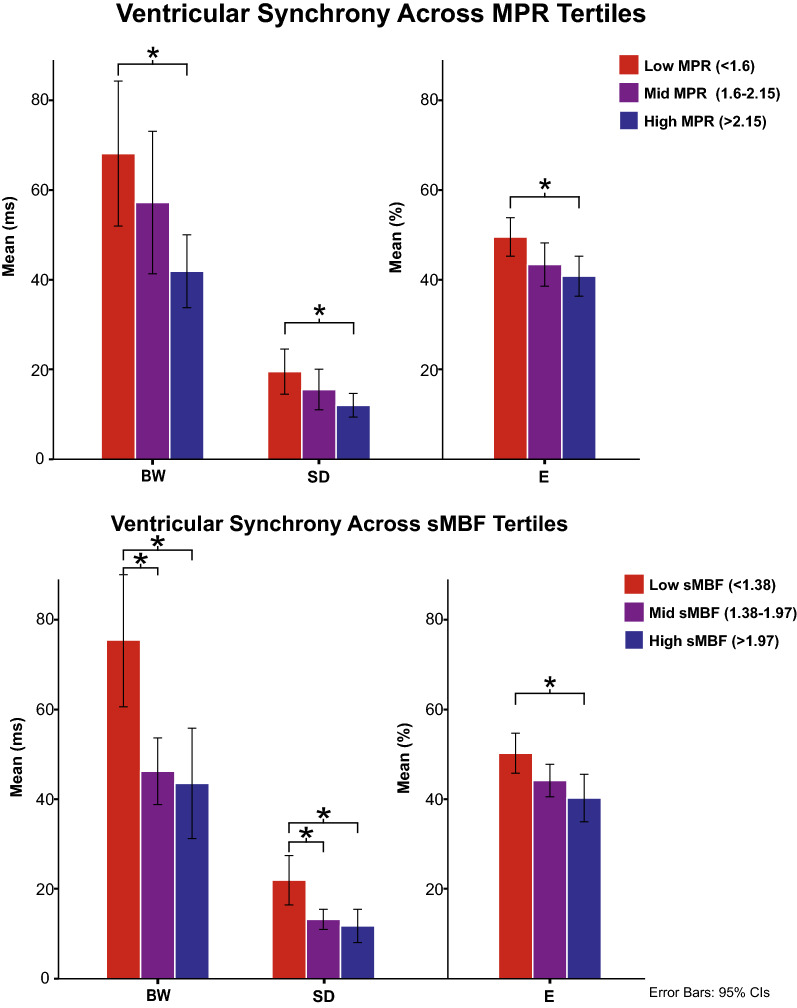
Bar chart depicting mean ventricular synchrony parameters (BW, SD, and E) across statistically defined MPR (upper chart) and sMBF (lower chart) tertiles. *p value < .5 for a post hoc corrected pairwise comparison

There were significant differences in peak stress ventricular mechanical synchrony parameters across myocardial perfusion variable tertiles documenting a significant inversely proportional perfusion-synchrony relationship. Notably, these differences were more pronounced across tertiles of sMBF than of MPR according to the calculated effect sizes (BW_η2 _= 0.17 vs 0.08, SD_η2 _= 0.15 vs 0.07, and E_η2 _= 0.10 vs 0.08). Significant pairwise differences are graphically depicted in Figure 1 and absolute numerical differences can be consulted in the supplementary material—Online Resource 1.

### Biserial Correlations Between Dependent Variables

We documented significant and strong correlations between mechanical ventricular synchrony parameters. Pearson’s correlation coefficients showed significance between all permutations of the considered synchrony parameters. These results are depicted in the correlation matrix shown in Table 2.

**Table 2:** Biserial correlations between dependent variables

	BW	SD	E
BW	1	0.96*	0.78*
SD	0.96*	1	0.77*
E	0.78*	0.77*	1

*BW*, bandwidth; *E*, entropy; *SD*, standard deviation

*p value < .001

### Stepwise Multivariate Analysis

**Figure 2:**
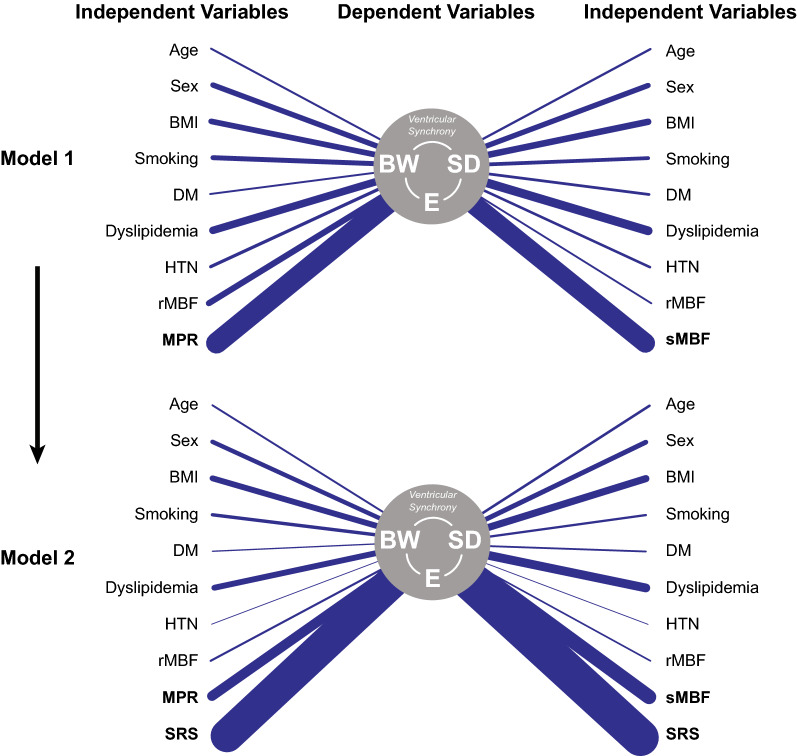
Pictorial depiction of the MANCOVA adjusted effect sizes for all evaluated predictors across models 1 and 2. The correlational lines between individual predictors and dependent variables are weighted according to their magnitude (see Online Resource 2)

A comprehensive pictorial representation of the MANCOVA results for all models is shown in Figure 2. The MANCOVA *Model 1* documented that MPR (*p*<.01) is an independent predictor of stress ventricular mechanical synchrony accounting for a medium effect size (η^2^= 0.229), while dyslipidemia only demonstrated a trend towards significance and a rather discrete effect (*p* = .09, η^2^ = 0.081) and rMBF did not document a significant effect (*p* = .17). Further, when SRS was included into *Model 2*, MPR was no longer a significant predictor of the outcome variables. Conversely, SRS was found to be the strongest independent predictor of mechanical synchrony variables demonstrating a considerably higher absolute effect size (*p* < .01, η^2^ = 0.358) than the other considered predictors.

**Figure 3:**
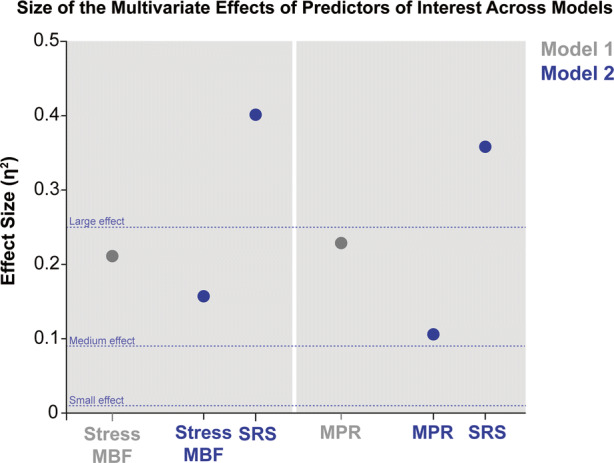
Point chart depicting the magnitude of the multivariate adjusted effect sizes of quantitative PET myocardial perfusion parameters (MPR and sMBF) in Model 1 and Model 2 (SRS is depicted only in comparison to perfusion parameters for Model 2)

When the analyses were repeated considering sMBF as the quantitative myocardial perfusion variable, results were similar. In *Model 1*, dyslipidemia only demonstrated a trend towards a significant effect (*p* = .06), while sMBF (*p* = .01) was a significant predictor of stress mechanical synchrony variables. Conversely, SRS overshadowed (once again) sMBF as the strongest independent predictor (*p* < .01, η^2^ = 0.401) in *Model 2*. Consistently, rMBF did not demonstrate a significant effect in the models (*p* = .70 and *p* = .74 in Model 1 and 2, respectively). Figure 3 provides an isolated view of the behavior of the effect sizes of the strongest predictors of interest across models.

The complementary results considering the perfusion and synchrony estimates at the coronary vessel-territory level demonstrated a similar behavior with SRS accounting for the predominating effect in the models. Two particular differences found in the supplementary analysis were (1) once SRS was included in the model, stress MBF and MPR showed a marginal trend towards significance in contrast with the global analysis (*p* = .06 and *p* = .07, respectively), and (2) rest MBF demonstrated a significant effect in *Model 1* that included MPR as the perfusion parameter.

These expanded numerical results can be consulted in the supplementary material—Online Resources 2 and 3.

## Discussion

The present study has shown that, in patients with chronic HF, absolute quantitative PET myocardial perfusion estimates are not independent determinants of ventricular mechanical synchrony when accounting for demographic characteristics, cardiovascular risk factors, and importantly, for the presence and extent of fixed myocardial perfusion defects. Conversely, it was documented that the SRS, as a surrogate of such fixed defects, represents the strongest independent predictor of PET-measured peak stress ventricular mechanical synchrony parameters.

In principle, it is understood that absolute myocardial perfusion status may be linked with the particular aspect of ventricular function objectified through the evaluation of mechanical synchrony. In fact, this notion has been investigated in both SPECT^23^ and PET^14^ imaging, and results have suggested that perfusion estimates convey a relevant association with mechanical synchrony and that this relationship likely interplays with risk factors and other aspects of myocardial status such as ventricular composition or architecture. Our study advances this concept by evaluating the comparative relevance of the most commonly used quantitative myocardial perfusion estimates (MPR and sMBF) as determinants of an integral axis of mechanical synchrony (provided by 3 related variables) during peak stress in the particular setting of chronic HF patients.

Based on our results, it became clear that even though the exploratory unadjusted comparisons of synchrony parameters (BW, SD, and E) across worsening MPR and sMBF tertiles showed significant differences, the adjusted (multivariate) analysis demonstrated that SRS conveys the strongest independent association with the outcome variables, overshadowing the effect of quantitative perfusion parameters. Although this analysis may raise the uncertainty of whether such effect originates from a tight relationship between SRS and quantitative perfusion variables (as patients with fixed perfusion defects will undoubtedly present a decrease in myocardial perfusion in absolute terms), the exploration of the SRS-MPR and SRS-sMBF correlations informed that collinearity did not hinder our analysis (*r* = −0.23 and − 0.41, respectively, not shown in the results).

An interesting finding in the univariate analysis was that ventricular synchrony parameters were considerably more sensitive to worsening values of sMBF than those of MPR. This collateral finding partially supports the perception suggested in previous reports of the superiority of sMBF as an absolute PET myocardial perfusion parameter.^14^,^24^ Furthermore, this larger effect of sMBF reflected into the fully adjusted multivariate analysis where a trend towards a significant association with ventricular synchrony was noticed (*p* = .06), contrary to the multivariate results considering MPR (*p* = .23). We believe that these effects become readily patent in the pictorial representation of the MACONVA models’ results. The comparative weight of the correlation lines underscores the relevance of perfusion parameters while also demonstrating the *major* role of fixed perfusion defects. Of note, even though rMBF was considered in the reported analyses, as it may be the case that this parameter is found affected in the particular set of patients with HF, the univariate and multivariate estimates of effect did not support a major influence of this perfusion parameter on mechanical synchrony.

Patients with HF show lower quantitative myocardial perfusion estimates^25^ and it is hypothesized that, among other factors, endothelial dysfunction may play a role in this phenomenon.^26^ Our results fall in line with this previous finding and it is possible that a blunted vasodilatory response could downsize the proposed association between perfusion and mechanical synchrony. In any case, we believe that a sober interpretation of our results gives reason to suggest that within the scenario of patients with chronic HF and although optimization of myocardial perfusion (including possible microvascular dysfunction) should represent a relevant part of the therapeutic approach, it may be more convenient to concentrate on the characterization of the fixed perfusion defects in terms of their presence, extent, severity, and probably even tissue composition overall (in which case CMR can offer great advantages) in order to improve the management of arguably relevant ventricular mechanical dyssynchrony.

A particular feature of our report is the incorporation and characterization of effect sizes in our interpretation of results. In fact, both the pictorial and numerical effect sizes provided by the multivariate analyses attest the comparative relevance of quantitative perfusion and of the presence/extent of fixed defects by demonstrating that although SRS was clearly the strongest independent predictor of synchrony (with a large effect), the magnitude of the effect sizes of MPR and sMBF were the second largest overall and mostly fall in the category of medium effects.

Fixed perfusion defects can be caused by a previous MI, but in our sample only 40% of patients had a medical history of MI. Still, all patients had the associated diagnosis of chronic HF and were referred to PET due to suspected (chronic) ischemia. Although myocardial scarring after major events such as MI is generally considered in the horizon of HF of suspected ischemic origin, it is also considered that physiopathological levels of chronic ischemia (probably at the microvascular level) can negatively influence myocardial architecture and ultimately its function. Moreover, it is also understood that a discrete proportion of MI events can be overlooked due to atypical symptomatology such as in diabetic patients and expectedly, the prevalence of DM in our sample was substantial. Consequently, we aimed to account for existing fixed perfusion defects rather than only on the history of previous MI. This means that, while we do not account for the particular origin or composition of the documented fixed perfusion defects, one can still argue that such non-reversible defects seem to be by far more influential on the integral axis of mechanical synchrony than the quantitative myocardial perfusion status obtained in absolute terms with PET. In fact, this notion was sustained when the analysis was repeated at a per-vessel territory (i.e., regional) level. Of course, we still believe that a regional analysis is relevant for the particular exploration of suspected localized ischemia and in the characterization of potentially viable residual tissue.

Nitrogen-13 ammonia PET allows for the evaluation of absolute myocardial perfusion and mechanical synchrony at real time-peak hyperemic stress, which confers an advantage over traditional post-stress SPECT acquisitions. Nevertheless, as non-invasive imaging advances the refinement of its implementations, we believe that multimodality imaging (considering other powerful techniques such as CMR) will benefit patient classification optimization by providing complementary information that, in the case of HF and its management, is currently incomplete.

Finally, it is worth considering that either if mechanical synchrony is viewed as a marker of disease,^27^,^28^ a risk factor,^4^ or as an addressable substrate for therapy (CRT),^29^ further understanding of the hierarchical structure of its determinants is desirable, and we consider that the present report is a discrete contribution to this horizon.

## Limitations

The present study carries all the limitations of a retrospective analysis. Furthermore, we have mentioned that the mechanistic nature of the fixed perfusion defects was not accounted for in every case and it is possible that a small proportion of artifacts may have factored into the SRS estimates. However, this may represent a minor issue given the superior quality of PET imaging when compared with traditional SPECT. Another limitation may be found in the sample size and constitution, it could be possible that the significance of perfusion estimates as determinants of ventricular synchrony was attenuated by the number of subjects included and by a mixture of possible etiologies. Yet, this report studied patients with the commonality of clinically-classified chronic HF evaluated in our institution by a dedicated clinic. Moreover, in the case of this sample, patients were referred for PET imaging when there was doubt of whether an ischemic component was present beyond the assumed etiology. Finally, our results have included effect sizes for the studied relations and we exhort the reader to bear such estimates in mind given their stability against sample size differences.

## Conclusions

The existence and extent of fixed perfusion defects, but not the quantitative PET myocardial perfusion parameters (sMBF and MPR), constitute a significant independent predictor of resulting peak-stress ventricular mechanical synchrony in patients with chronic HF.

## New Knowledge Gained

Among patients with chronic HF referred to Nitrogen-13 ammonia PET/CT imaging, the presence and extent of pre-existing fixed perfusion defects, but not quantitative myocardial perfusion parameters, constitute a significant independent determinant of resulting peak-stress ventricular mechanical synchrony.

## Electronic supplementary material

Below is the link to the electronic supplementary material.

Supplementary material 1 (DOCX 24 kb)

Supplementary material 2 (PPTX 320 kb)

## Abbreviations

BW: Bandwidth
MBF: Myocardial blood flow
MI: Myocardial infarction
MPR: Myocardial perfusion reserve
SD: Standard deviation
SRS: Summed rest score
E: Entropy
HF: Heart failure

## References

[1] Ambrosy AP, Fonarow GC, Butler J, Chioncel O, Greene SJ, Vaduganathan M (2014). The global health and economic burden of hospitalizations for heart failure: Lessons learned from hospitalized heart failure registries. J Am Coll Cardiol.

[2] De Luca L, Marini M, Gonzini L, Boccanelli A, Casella G, Chiarella F (2016). Contemporary trends and age-specific sex differences in management and outcome for patients with ST-segment elevation myocardial infarction. J Am Heart Assoc..

[3] McMurray JJV (2010). Clinical practice. Systolic heart failure. N Engl J Med.

[4] Shah AM, Solomon SD (2016). Mechanical dyssynchrony: A risk factor but not a target. Eur Heart J.

[5] Tang ASL, Wells GA, Talajic M, Arnold MO, Sheldon R, Connolly S (2010). Cardiac-resynchronization therapy for mild-to-moderate heart failure. N Engl J Med.

[6] European Society of Cardiology (ESC), European Heart Rhythm Association (EHRA), Brignole M, Auricchio A, Baron-Esquivias G, Bordachar P, et al. (2013) ESC guidelines on cardiac pacing and cardiac resynchronization therapy: the task force on cardiac pacing and resynchronization therapy of the European Society of Cardiology (ESC). Developed in collaboration with the European Heart Rhythm Association. Europace. 2013;15:1070-118 10.1093/europace/eut206 23801827

[7] Young JB, Abraham WT, Smith AL, Leon AR, Lieberman R, Wilkoff B (2003). Combined cardiac resynchronization and in advanced chronic heart failure. Am Med Assoc.

[8] Abraham WT, Fisher WG, Smith AL, Delurgio DB, Leon AR, Loh E (2002). Cardiac resynchronization in chronic heart failure. N Engl J Med.

[9] Slart RHJA, Glauche J, Golestani R, Zeebregts CJ, Jansen JW, Dierckx RAJO (2012). PET and MRI for the evaluation of regional myocardial perfusion and wall thickening after myocardial infarction. Eur J Nucl Med Mol Imaging.

[10] Juárez-Orozco LE, Glauche J, Alexanderson E, Zeebregts CJ, Boersma HH, Glaudemans AWJM (2013). Myocardial perfusion reserve in spared myocardium: Correlation with infarct size and left ventricular ejection fraction. Eur J Nucl Med Mol Imaging.

[11] Juarez-Orozco LE, Cruz-Mendoza JR, Guinto-Nishimura GY, Walls-Laguarda L, Casares-Echeverría LJ, Meave-Gonzalez A (2018). PET myocardial perfusion quantification: Anatomy of a spreading functional technique. Clin Transl Imaging.

[12] Chen J, Faber TL, Cooke CD, Garcia EV (2008). Temporal resolution of multiharmonic phase analysis of ECG-gated myocardial perfusion SPECT studies. J Nucl Cardiol.

[13] Huang W-S, Huang C-H, Lee C-L, Chen C-P, Hung G-U, Chen J (2014). Relation of early post-stress left ventricular dyssynchrony and the extent of angiographic coronary artery disease. J Nucl Cardiol.

[14] Juárez-Orozco LE, Alexanderson E, Dierckx RA, Boersma HHH, Hillege JL, Zeebregts CJ (2016). Stress myocardial blood flow correlates with ventricular function and synchrony better than myocardial perfusion reserve: A Nitrogen-13 ammonia PET study. J Nucl Cardiol.

[15] Opstal TSJ, Knol RJJ, Cornel JH, Wondergem M, van der Zant FM (2018). Myocardial blood flow and myocardial flow reserve values in 13N-ammonia myocardial perfusion PET/CT using a time-efficient protocol in patients without coronary artery disease. Eur J Hybrid Imaging.

[16] Hutchins GD, Schwaiger M, Rosenspire KC, Krivokapich J, Schelbert H, Kuhl DE (1990). Noninvasive quantification of regional blood flow in the human heart using N-13 ammonia and dynamic positron emission tomographic imaging. J Am Coll Cardiol.

[17] Cerqueira MD, Weissman NJ, Dilsizian V, Jacobs AK, Kaul S, Laskey WK (2002). Standardized myocardial segmentation and nomenclature for tomographic imaging of the heart. A statement for healthcare professionals from the Cardiac Imaging Committee of the Council on Clinical Cardiology of the American Heart Association. Circulation.

[18] Chow BJW, Dorbala S, Di Carli MF, Merhige ME, Williams BA, Veledar E (2014). Prognostic value of PET myocardial perfusion imaging in obese patients. JACC Cardiovasc Imaging.

[19] Sharir T, Germano G, Kavanagh PB, Lai S, Cohen I, Lewin HC (1999). Incremental prognostic value of post-stress left ventricular ejection fraction and volume by gated myocardial perfusion single photon emission computed tomography. Circulation.

[20] Chen J, Garcia EV, Folks RD, Cooke CD, Faber TL, Tauxe EL (2005). Onset of left ventricular mechanical contraction as determined by phase analysis of ECG-gated myocardial perfusion SPECT imaging: Development of a diagnostic tool for assessment of cardiac mechanical dyssynchrony. J Nucl Cardiol.

[21] Uebleis C, Ulbrich M, Tegtmeyer R, Schuessler F, Haserueck N, Siebermair J (2011). Electrocardiogram-gated 18F-FDG PET/CT hybrid imaging in patients with unsatisfactory response to cardiac resynchronization therapy: Initial clinical results. J Nucl Med.

[22] LE Juárez-Orozco, Tio RA, Alexanderson E, Dweck M, Vliegenthart R, El Moumni M (2018). Quantitative myocardial perfusion evaluation with positron emission tomography and the risk of cardiovascular events in patients with coronary artery disease: A systematic review of prognostic studies. Eur Heart J Cardiovasc Imaging.

[23] Chen C-C, Shen T-Y, Chang M-C, Hung G-U, Chen W-C, Kao C-H (2012). Stress-induced myocardial ischemia is associated with early post-stress left ventricular mechanical dyssynchrony as assessed by phase analysis of 201Tl gated SPECT myocardial perfusion imaging. Eur J Nucl Med Mol Imaging.

[24] Joutsiniemi E, Saraste A, Pietilä M, Mäki M, Kajander S, Ukkonen H (2014). Absolute flow or myocardial flow reserve for the detection of significant coronary artery disease?. Eur Heart J Cardiovasc Imaging.

[25] Srivaratharajah K, Coutinho T, DeKemp R, Liu P, Haddad H, Stadnick E (2016). Reduced myocardial flow in heart failure patients with preserved ejection fraction. Circ Heart Fail.

[26] De Boer RA, Pinto YM, Van Veldhuisen DJ (2003). The imbalance between oxygen demand and supply as a potential mechanism in the pathophysiology of heart failure: The role of microvascular growth and abnormalities. Microcirculation.

[27] AlJaroudi W (2014). Early post-stress LV dyssynchrony: A new marker for significant CAD. J Nucl Cardiol.

[28] Van Tosh A, Votaw JR, Cooke CD, Reichek N, Palestro CJ, Nichols KJ (2017). Relationships between left ventricular asynchrony and myocardial blood flow. J Nucl Cardiol.

[29] Murrow J, Esteves F, Galt J, Chen J, Garcia E, Lin J (2011). Characterization of mechanical dyssynchrony measured by gated single photon emission computed tomography phase analysis after acute ST-elevation myocardial infarction. J Nucl Cardiol.

