# Supplementary material for: Ventricular synchrony is not significantly determined by absolute myocardial perfusion in patients with chronic heart failure: A 13N-ammonia PET study
Source: J Nucl Cardiol. 2018 Nov 15;27(6):2234–42. doi: 10.1007/s12350-018-01507-9 (PMC7749096; doi:10.1007/s12350-018-01507-9)
Supplement: Supplementary file 1 — Supplementary material 1 (DOCX 24 kb) [file 12350_2018_1507_MOESM1_ESM.docx]

**ONLINE SUPPLEMENTARY MATERIAL**

**Title:** Ventricular synchrony is not significantly determined by absolute myocardial perfusion in patients with chronic heart failure: A 13N-ammonia PET study

**Journal:** Journal of Nuclear Cardiology

**Authors:** Luis Eduardo Juarez-Orozco MD PhD, Andrea G Monroy-Gonzalez MD, Friso M van der Zant MD PhD, Nick Hoogvorst, Riemer HJA Slart MD PhD, Remco JJ Knol MD PhD

**Corresponding Author:** *Luis Eduardo Juarez-Orozco, MD, PhD. Department of Nuclear Medicine and Molecular Imaging, University Medical Center Groningen, The Netherlands.*

*Email:* [*l.e.juarez.orozco@gmail.com*](mailto:l.e.juarez.orozco@gmail.com)

**Online Resource 1.** Univariate ANOVA analyses of mechanical synchrony parameters across perfusion tertiles

|  | CFR tertiles | | |  |  |
| --- | --- | --- | --- | --- | --- |
|  | ***Low*** | ***Middle*** | ***High*** | ***p-value*** | ***η^2^*** |
| BW (ms) | 68.9 (44.6) | 57.9 (43.8) | 42.4 (22.5) | **0.027** | 0.077 |
| SD (ms) | 19.7 (13.9) | 15.6 (12.5) | 12.1 (7.3) | **0.039** | 0.069 |
| E (%) | 49.9 (11.8) | 43.8 (13.3) | 41.2 (12.3) | **0.020** | 0.084 |
|  | **sMBF tertiles** | | |  |  |
|  | ***Low*** | ***Middle*** | ***High*** | ***p-value*** | ***η^2^*** |
| BW (ms) | 78.9 (49.4) | 46.5 (20.3) | 43.7 (33.6) | **<0.001** | 0.166 |
| SD (ms) | 22.1 (15.0) | 13.4 (6.2) | 11.9 (10.2) | **0.001** | 0.147 |
| E (%) | 50.4 (12.2) | 44.3 (9.9) | 40.3 (14.5) | **0.007** | 0.104 |

**Online Resource 2.** MANCOVA analyses of predictors of stress ventricular mechanical synchrony

| A | Model 1 | | | | Model 2 | | | |
| --- | --- | --- | --- | --- | --- | --- | --- | --- |
| Dependent Var | **Independent Var** | **Pillai's Trace (and η^2^)** | **F** | ***p*** | **Independent Var** | **Pillai's Trace (and η^2^)** | **F** | ***p*** |
| BW  SD  E | Intercept | 0.220 | 7.055 | 0.00 | Intercept | 0.111 | 3.047 | 0.03 |
|  | Age | 0.023 | 0.588 | 0.62 | Age | 0.020 | 0.504 | 0.68 |
|  | Sex | 0.058 | 1.536 | 0.21 | Sex | 0.047 | 1.198 | 0.32 |
|  | BMI | 0.061 | 1.615 | 0.19 | BMI | 0.063 | 1.634 | 0.19 |
|  | Smoking | 0.053 | 1.399 | 0.25 | Smoking | 0.036 | 0.899 | 0.45 |
|  | DM | 0.020 | 0.513 | 0.67 | DM | 0.013 | 0.315 | 0.81 |
|  | Dyslipidemia | 0.081 | 2.202 | 0.09 | Dyslipidemia | 0.063 | 1.626 | 0.19 |
|  | HTN | 0.034 | 0.875 | 0.46 | HTN | 0.004 | 0.097 | 0.96 |
|  | rMBF | 0.065 | 1.727 | 0.17 | rMBF | 0.022 | 0.545 | 0.65 |
|  | **MPR (tertiles)** | **0.229** | **3.270** | **<0.01** | **MPR (tertiles)** | 0.106 | 1.375 | 0.23 |
|  |  |  |  |  | **SRS** | **0.358** | **13.542** | **<0.01** |
|  |  |  |  |  |  |  |  |  |
| B | Model 1 | | | | Model 2 | | | |
| Dependent Var | **Independent Var** | **Pillai's Trace (and η^2^)** | **F** | ***p*** | **Independent Var** | **Pillai's Trace (and η^2^)** | **F** | ***p*** |
| BW  SD  E | Intercept | 0.132 | 3.806 | 0.01 | Intercept | 0.056 | 1.442 | 0.24 |
|  | Age | 0.024 | 0.604 | 0.61 | Age | 0.027 | 0.688 | 0.56 |
|  | Sex | 0.057 | 1.511 | 0.22 | Sex | 0.052 | 1.34 | 0.27 |
|  | BMI | 0.070 | 1.868 | 0.14 | BMI | 0.077 | 2.022 | 0.12 |
|  | Smoking | 0.040 | 1.051 | 0.38 | Smoking | 0.024 | 0.592 | 0.62 |
|  | DM | 0.029 | 0.735 | 0.53 | DM | 0.019 | 0.472 | 0.70 |
|  | Dyslipidemia | 0.095 | 2.621 | 0.06 | Dyslipidemia | 0.095 | 2.559 | 0.06 |
|  | HTN | 0.027 | 0.701 | 0.55 | HTN | 0.007 | 0.17 | 0.92 |
|  | rMBF | 0.019 | 0.481 | 0.70 | rMBF | 0.017 | 0.425 | 0.74 |
|  | **sMBF (tertiles)** | **0.211** | **2.982** | **0.01** | **sMBF (tertiles)** | 0.157 | 2.098 | 0.06 |
|  |  |  |  |  | **SRS** | **0.401** | **16.321** | **<0.01** |

**Online Resource 3.** MANCOVA per-vessel territory analyses of predictors of stress ventricular mechanical synchrony

| A | Model 1 | | | | Model 2 | | | |
| --- | --- | --- | --- | --- | --- | --- | --- | --- |
| Dependent Var | **Independent Var** | **Pillai's Trace (and η^2^)** | **F** | ***p*** | **Independent Var** | **Pillai's Trace (and η^2^)** | **F** | ***p*** |
| BW  SD  E | Intercept | 0.116 | 10.904 | 0.00 | Intercept | 0.039 | 3.374 | 0.02 |
|  | Age | 0.002 | 0.145 | 0.93 | Age | 0.006 | 0.497 | 0.68 |
|  | Sex | 0.021 | 1.769 | 0.15 | Sex | 0.017 | 1.434 | 0.23 |
|  | BMI | 0.016 | 1.342 | 0.26 | BMI | 0.015 | 1.226 | 0.30 |
|  | Smoking | 0.020 | 1.67 | 0.17 | Smoking | 0.016 | 1.311 | 0.27 |
|  | DM | 0.004 | 0.326 | 0.81 | DM | 0.009 | 0.735 | 0.53 |
|  | Dyslipidemia | 0.019 | 1.587 | 0.19 | Dyslipidemia | 0.016 | 1.379 | 0.25 |
|  | HTN | 0.016 | 1.334 | 0.26 | HTN | 0.002 | 0.17 | 0.92 |
|  | **rMBF** | **0.060** | **5.331** | **<0.01** | rMBF | 0.022 | 1.83 | 0.14 |
|  | **MPR** | **0.081** | **7.33** | **<0.01** | MPR | 0.028 | 2.345 | 0.07 |
|  |  |  |  |  | **SRS** | **0.139** | **13.348** | **<0.01** |
|  |  |  |  |  |  |  |  |  |
| B | Model 1 | | | | Model 2 | | | |
| Dependent Var | **Independent Var** | **Pillai's Trace (and η^2^)** | **F** | ***p*** | **Independent Var** | **Pillai's Trace (and η^2^)** | **F** | ***p*** |
| BW  SD  E | Intercept | 0.098 | 9.017 | 0.00 | Intercept | 0.037 | 3.219 | 0.02 |
|  | Age | 0.001 | 0.094 | 0.96 | Age | 0.006 | 0.521 | 0.67 |
|  | Sex | 0.019 | 1.626 | 0.18 | Sex | 0.018 | 1.545 | 0.20 |
|  | BMI | 0.016 | 1.313 | 0.27 | BMI | 0.014 | 1.182 | 0.32 |
|  | Smoking | 0.018 | 1.531 | 0.21 | Smoking | 0.015 | 1.221 | 0.30 |
|  | DM | 0.004 | 0.368 | 0.78 | DM | 0.010 | 0.812 | 0.49 |
|  | Dyslipidemia | 0.019 | 1.607 | 0.19 | Dyslipidemia | 0.015 | 1.27 | 0.29 |
|  | HTN | 0.015 | 1.273 | 0.28 | HTN | 0.002 | 0.206 | 0.89 |
|  | rMBF | 0.001 | 0.071 | 0.98 | rMBF | 0.001 | 0.078 | 0.97 |
|  | **sMBF** | **0.088** | **8.017** | **0.00** | sMBF | 0.030 | 2.537 | 0.06 |
|  |  |  |  |  | **SRS** | **0.134** | **12.839** | **0.00** |
