# Supplementary material for: Ventricular synchrony is not significantly determined by absolute myocardial perfusion in patients with chronic heart failure: A 13N-ammonia PET study
Source: J Nucl Cardiol. 2018 Nov 15;27(6):2234–42. doi: 10.1007/s12350-018-01507-9 (PMC7749096; doi:10.1007/s12350-018-01507-9)
Supplement: Supplementary file 2 — Supplementary material 2 (PPTX 320 kb) [file 12350_2018_1507_MOESM2_ESM.pptx]

## Slide 1
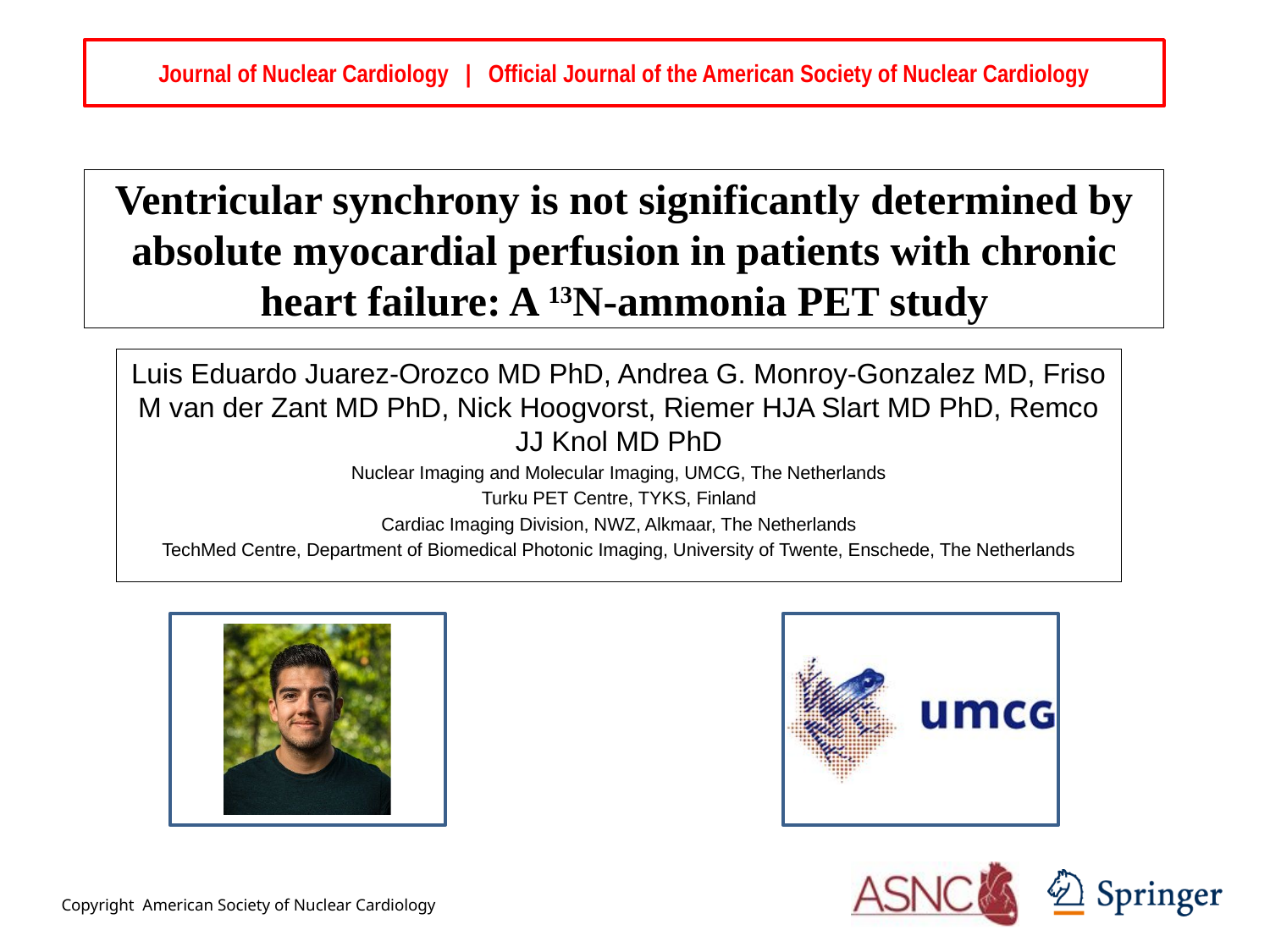

Journal of Nuclear Cardiology | Official Journal of the American Society of Nuclear Cardiology
# Ventricular synchrony is not significantly determined by absolute myocardial perfusion in patients with chronic heart failure: A 13N-ammonia PET study
Luis Eduardo Juarez-Orozco MD PhD, Andrea G. Monroy-Gonzalez MD, Friso M van der Zant MD PhD, Nick Hoogvorst, Riemer HJA Slart MD PhD, Remco JJ Knol MD PhD
Nuclear Imaging and Molecular Imaging, UMCG, The Netherlands
Turku PET Centre, TYKS, Finland
Cardiac Imaging Division, NWZ, Alkmaar, The Netherlands
TechMed Centre, Department of Biomedical Photonic Imaging, University of Twente, Enschede, The Netherlands
Copyright American Society of Nuclear Cardiology

## Slide 2
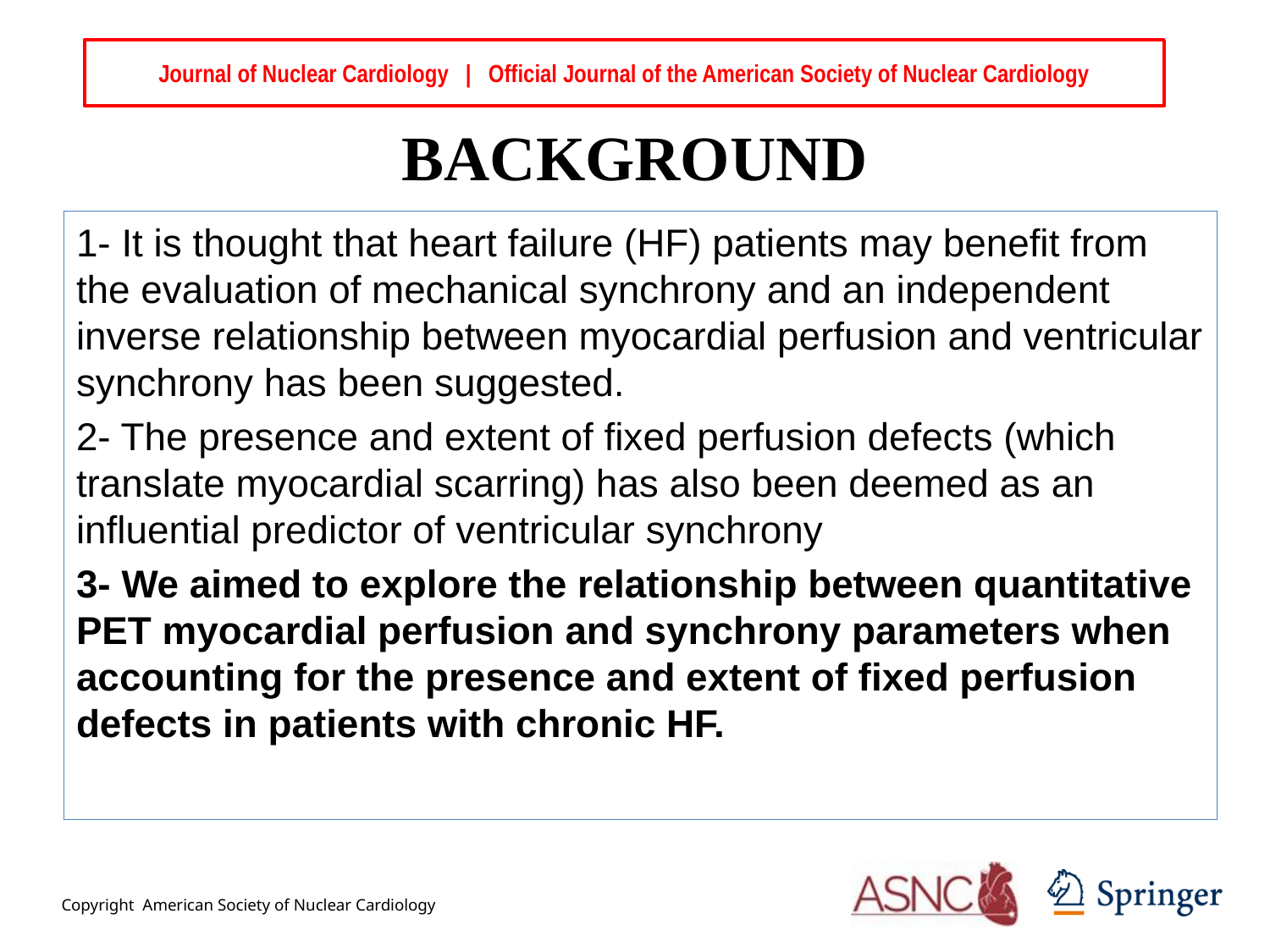

Journal of Nuclear Cardiology | Official Journal of the American Society of Nuclear Cardiology
# BACKGROUND
1- It is thought that heart failure (HF) patients may benefit from the evaluation of mechanical synchrony and an independent inverse relationship between myocardial perfusion and ventricular synchrony has been suggested.
2- The presence and extent of fixed perfusion defects (which translate myocardial scarring) has also been deemed as an influential predictor of ventricular synchrony
3- We aimed to explore the relationship between quantitative PET myocardial perfusion and synchrony parameters when accounting for the presence and extent of fixed perfusion defects in patients with chronic HF.
Copyright American Society of Nuclear Cardiology

## Slide 3
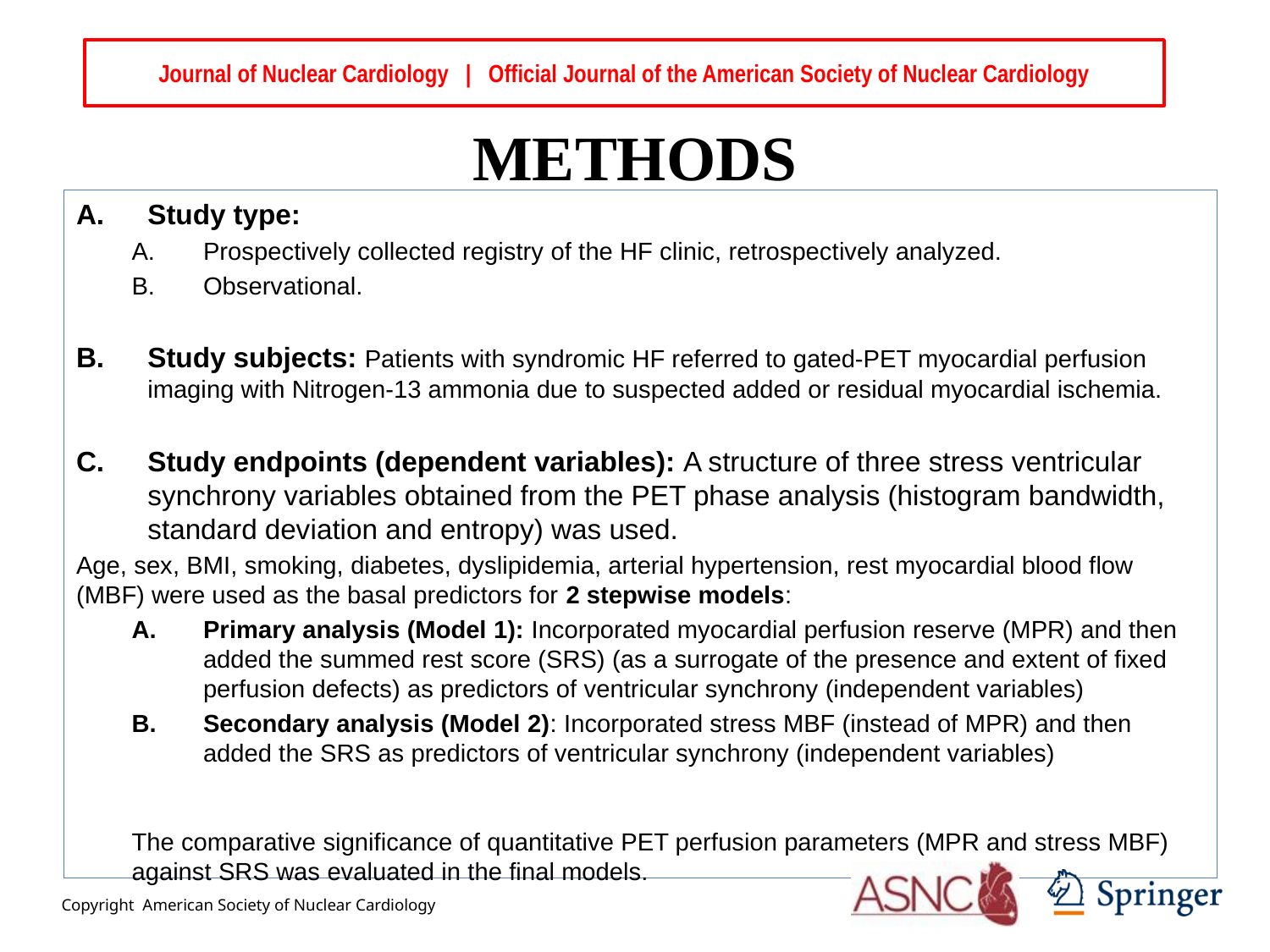

Journal of Nuclear Cardiology | Official Journal of the American Society of Nuclear Cardiology
# METHODS
Study type:
Prospectively collected registry of the HF clinic, retrospectively analyzed.
Observational.
Study subjects: Patients with syndromic HF referred to gated-PET myocardial perfusion imaging with Nitrogen-13 ammonia due to suspected added or residual myocardial ischemia.
Study endpoints (dependent variables): A structure of three stress ventricular synchrony variables obtained from the PET phase analysis (histogram bandwidth, standard deviation and entropy) was used.
Age, sex, BMI, smoking, diabetes, dyslipidemia, arterial hypertension, rest myocardial blood flow (MBF) were used as the basal predictors for 2 stepwise models:
Primary analysis (Model 1): Incorporated myocardial perfusion reserve (MPR) and then added the summed rest score (SRS) (as a surrogate of the presence and extent of fixed perfusion defects) as predictors of ventricular synchrony (independent variables)
Secondary analysis (Model 2): Incorporated stress MBF (instead of MPR) and then added the SRS as predictors of ventricular synchrony (independent variables)
The comparative significance of quantitative PET perfusion parameters (MPR and stress MBF) against SRS was evaluated in the final models.
Copyright American Society of Nuclear Cardiology

## Slide 4
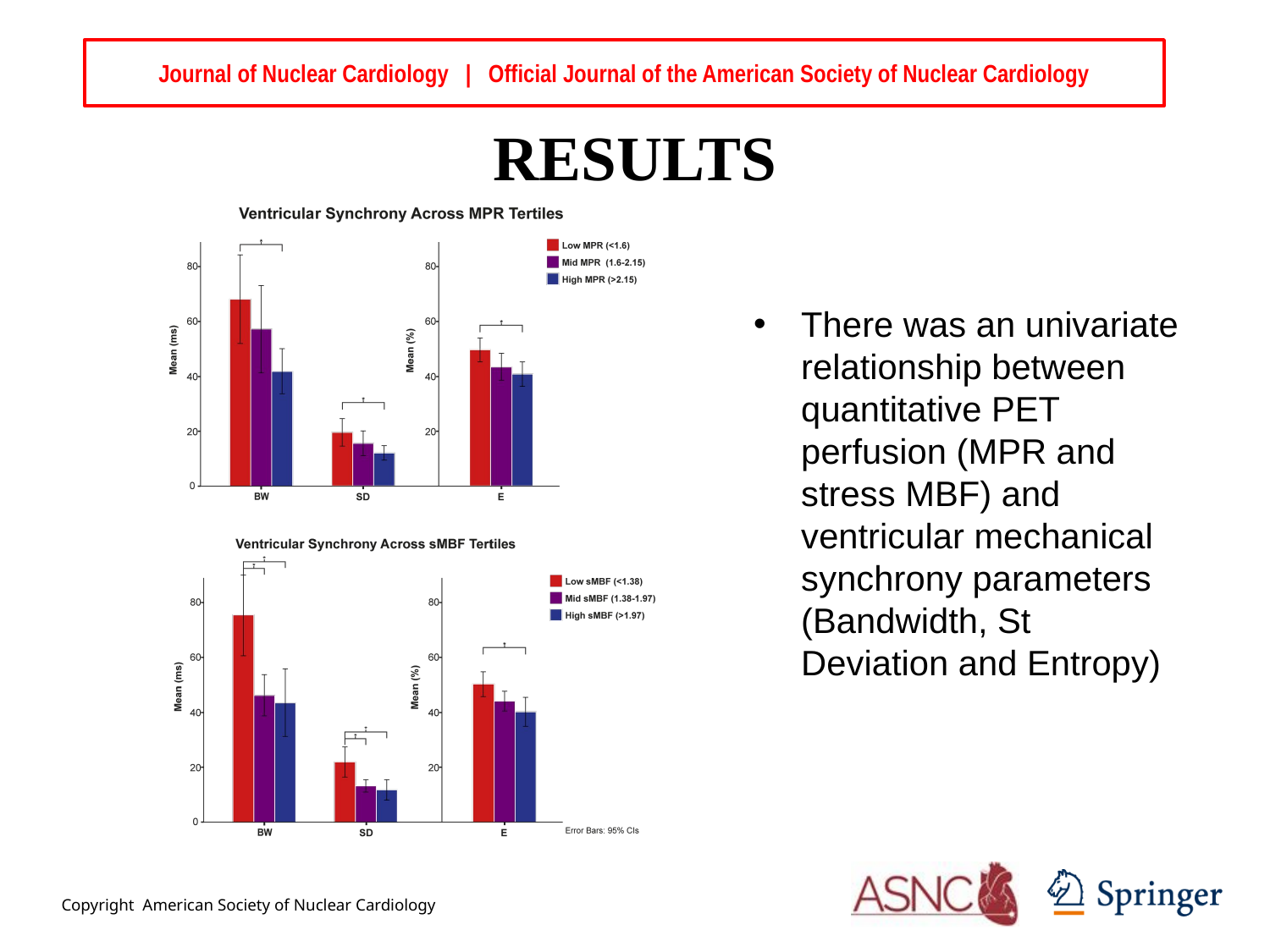

Journal of Nuclear Cardiology | Official Journal of the American Society of Nuclear Cardiology
# RESULTS
There was an univariate relationship between quantitative PET perfusion (MPR and stress MBF) and ventricular mechanical synchrony parameters (Bandwidth, St Deviation and Entropy)
Copyright American Society of Nuclear Cardiology

## Slide 5
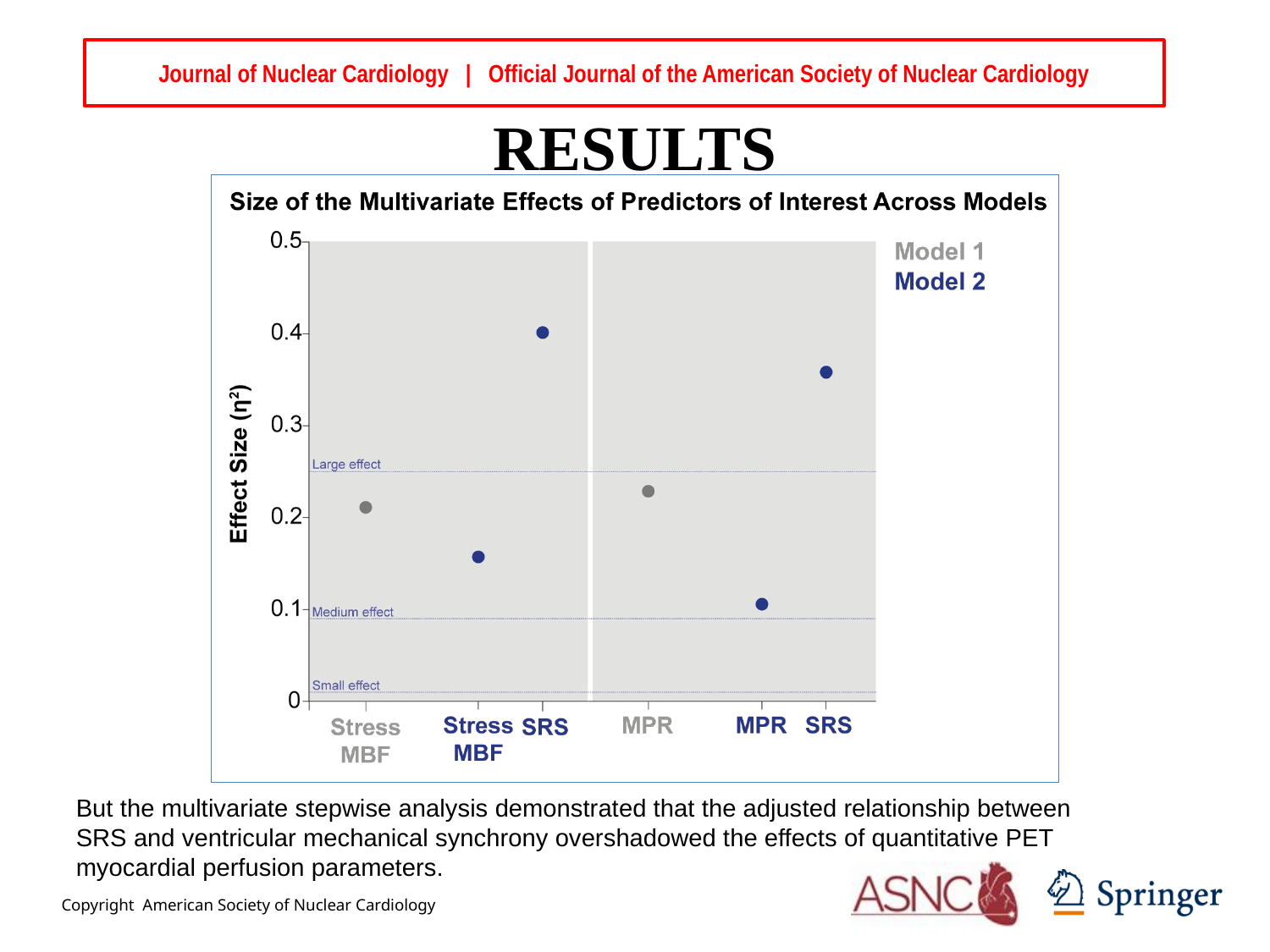

Journal of Nuclear Cardiology | Official Journal of the American Society of Nuclear Cardiology
# RESULTS
But the multivariate stepwise analysis demonstrated that the adjusted relationship between SRS and ventricular mechanical synchrony overshadowed the effects of quantitative PET myocardial perfusion parameters.
Copyright American Society of Nuclear Cardiology

## Slide 6
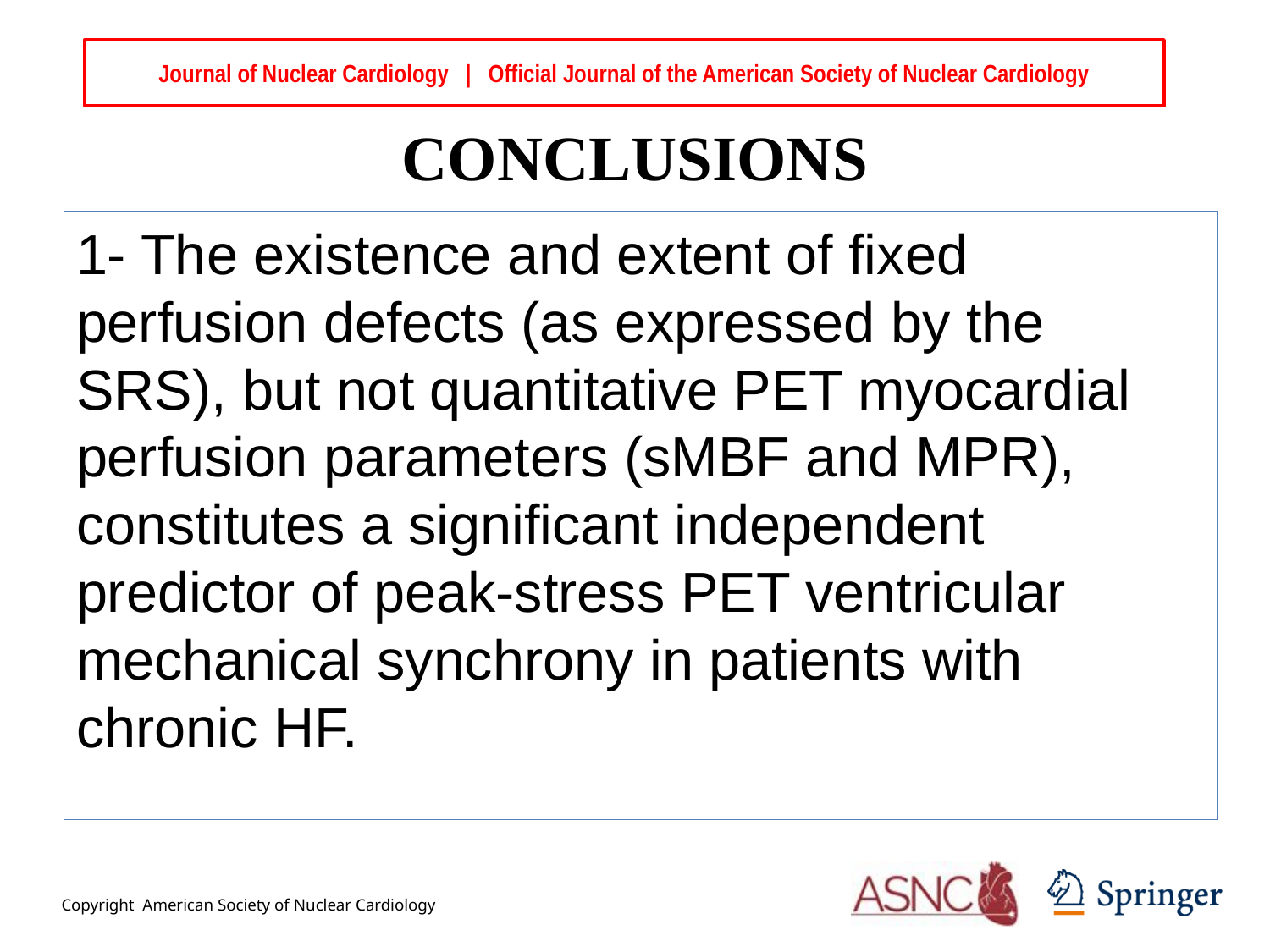

Journal of Nuclear Cardiology | Official Journal of the American Society of Nuclear Cardiology
# CONCLUSIONS
1- The existence and extent of fixed perfusion defects (as expressed by the SRS), but not quantitative PET myocardial perfusion parameters (sMBF and MPR), constitutes a significant independent predictor of peak-stress PET ventricular mechanical synchrony in patients with chronic HF.
Copyright American Society of Nuclear Cardiology
